# Towards Cancer Nanoradiopharmaceuticals—Radioisotope Nanocarrier System for Prostate Cancer Theranostics Based on Radiation-Synthesized Polymer Nanogels

**DOI:** 10.3390/cancers15235646

**Published:** 2023-11-29

**Authors:** Beata Paulina Rurarz, Kinga Anna Urbanek, Urszula Karczmarczyk, Joanna Raczkowska, Dominika Ewa Habrowska-Górczyńska, Marta Justyna Kozieł, Karolina Kowalska, Sławomir Kadłubowski, Agnieszka Sawicka, Michał Maurin, Agnieszka Wanda Piastowska-Ciesielska, Piotr Ulański

**Affiliations:** 1 Institute of Applied Radiation Chemistry, Faculty of Chemistry, Lodz University of Technology, Wroblewskiego 15, 93-590 Lodz, Polandslawomir.kadlubowski@p.lodz.pl (S.K.); piotr.ulanski@p.lodz.pl (P.U.); 2 Medical University of Lodz, Department of Cell Cultures and Genomic Analysis, Zeligowskiego 7/9, 90-752 Lodz, Poland; kinga.urbanek@umed.lodz.pl (K.A.U.); dominika.habrowska@umed.lodz.pl (D.E.H.-G.); marta.koziel@umed.lodz.pl (M.J.K.); karolina.kowalska1@umed.lodz.pl (K.K.); agnieszka.piastowska@umed.lodz.pl (A.W.P.-C.); 3 National Centre for Nuclear Research, Radioisotope Centre POLATOM, Andrzeja Soltana 7, 05-400 Otwock, Poland; urszula.karczmarczyk@polatom.pl (U.K.); agnieszka.sawicka@polatom.pl (A.S.); michal.maurin@polatom.pl (M.M.); 4 Medical University of Lodz, BRaIn Laboratories, Czechoslowacka 4, 92-216 Lodz, Poland

**Keywords:** prostate cancer, nanogels, nanocarriers, nanoradiopharmaceuticals, bombesin, gastrin-releasing peptide receptor, radiation synthesis, poly(acrylic acid), preclinical study

## Abstract

**Simple Summary:**

This research article reports the complete process of the fabrication and characterization of a targeted radioisotope nanocarrier platform designed for the theranostic management of prostate cancer. The state-of-the-art radiation synthesis of poly(acrylic acid) nanogels is followed by functionalization and radiolabeling, as well as both in vitro and in vivo assessment of the nanoplatform’s performance. Despite the need for further optimization of the system, our research is an outstanding example of radionanomedicine applications in the field of theranostic cancer management.

**Abstract:**

Despite the tremendous development of oncology, prostate cancer remains a debilitating malignancy. One of the most promising approaches to addressing this issue is to exploit the advancements of nanomedicine in combination with well-established nuclear medicine and radiotherapy. Following this idea, we have developed a radioisotope nanocarrier platform of electron-beam-synthesized nanogels based on poly(acrylic acid). We have developed a functionalization protocol, showing the very high (>97%) efficiency of the conjugation in targeting a ligand–bombesin derivative. This engineered peptide can bind gastrin-releasing peptide receptors overexpressed in prostate cancer cells; moreover, it bears a radioisotope-chelating moiety. Our nanoplatform exhibits very promising performance in vitro; the radiolabeled nanocarriers maintained high radiochemical purity of >90% in both the labeling buffer and human serum for up to 14 days. The application of the targeted nanocarrier allowed also effective and specific uptake in PC-3 prostate cancer cells, up to almost 30% after 4 h, which is a statistically significant improvement in comparison to carrier-free radiolabeled peptides. Although our system requires further studies for more promising results in vivo, our study represents a vital advancement in radionanomedicine—one of many steps that will lead to effective therapy for castration-resistant prostate cancer.

## 1. Introduction

Prostate cancer (PCa) is one of the leading causes of cancer death among men worldwide. Its incidence is associated with population aging and the growing income—in 112 countries, PCa is the most commonly diagnosed cancer in men [1]. It is considered that age, ethnicity, family factors, and environmental factors are proven risk factors for PCa. Obesity, diabetes mellitus, dietary patterns such as the Western diet, and low physical activity are proven environmental risk factors of PCa [2]. Although the usefulness of prostate-specific antigen (PSA) screening is still disputable, the five-year survival in PCa patients is reaching 100%. However, after radical prostatectomy, one third of patients present biochemical recurrence [3]. After the initial benefit from the therapy, many men will develop castration-resistant prostate cancer (CRPC), for which no effective therapy is available; thus, it constitutes a lethal malignancy—the median survival ratio amounts to 9 to 30 months [3].

The progress in understanding the molecular basis of PCa, as well as the cellular pathways involved in tumor development and progression, have led to substantial advancement in the field of targeted therapies, which offer promising tools in PCa management. An important part of this trend is the exploitation of advancements in the field of nanomedicine. It offers PCa patients targeted therapy by means of tailored drug delivery based on the avidity of specific ligands to the receptors overexpressed on tumor cells. One of these receptors, broadly recognized and studied in PCa [4], is the gastrin-releasing peptide receptor (GRPR). It is a member of the mammalian bombesin receptor family, one of the G-protein coupled receptors, performing a wide range of both physiological and pathophysiological functions by activating the phospholipase C signaling pathway [5]. GRPR is overexpressed in several malignancies, and, in tumor cells, it is predominantly involved in cell proliferation and cell cycle progression [6]. GRPR was found to be abundant not only in cell cultures in vitro, but also in patients’ cancer samples [7], thereby providing a rationale for its therapeutic and diagnostic value. Targeting GRPR in PCa cells is possible i.a. with a neuropeptide, bombesin, and its analogues, which have a homologous set of amino acids with mammalian gastrin-releasing peptide and competitively bind to its receptor and activate the downstream effects [5]. However, over years of research, many antagonistic ligands of GRPR have been discovered, which possess numerous advantages, such as efficient washout, a higher tumor/background ratio, and no side effects connected with GRPR activation [8]. Nevertheless, in most of the studies reported in the field of GRPR nanotargeting, agonists such as bombesin and its various derivatives are employed [4].

A particularly interesting application of the abovementioned concepts is radionanomedicine—an emerging field of science that combines the fundamentals of nuclear medicine and radiotherapy with the achievements of nanomedicine. Radiolabeled bombesin analogues, both agonists and antagonists, provide a range of possibilities for the imaging of prostate cancer in men. Nowadays, different bombesin analogues have been labeled with gamma (In-111 [9], Tc-99m [10]) and positron-emitting radionuclides (Ga-68 [11], F-18 [12] and Cu-64 [13]) for the imaging of GRPR-expressing tumors, in addition to cytotoxic beta (Lu-177 [10,14], Cu-67 [15]) and alpha particle-emitting nuclides (Bi-212/213 [16], Ac-225 [17]) for targeted radionuclide therapy. 

Integrating state-of-the-art nanocarriers with well-established radionuclides and specific targeting ligands can truly bring about change for patients suffering from PCa, as well as other life-threatening malignancies. Many research groups have exploited this area and numerous radionanosystems have been documented in the literature. Gibbens-Bandala et al. developed theranostic nanocarriers functionalized with bombesin and radioisotope-chelating 1,4,7,10-tetraazacyclododecane-1,4,7,10-tetraacetate (DOTA), based on PLGA nanoparticles [18], as well as PAMAM dendrimers [19]. They managed to show the promising performance of their systems both in vitro and in vivo in breast cancer models. Upon radiolabeling and intratumoral injections, the formulations reduced the size of the tumors; moreover, they were also shown to allow convenient SPECT imaging. PAMAM dendrimers have also been exploited as hybrid systems with gold nanoparticles encapsulated in their cavities. Mendoza-Nava functionalized such particles with multifunctional peptides comprising a GRPR-targeting DOTA–bombesin moiety, as well as folate for boosted targeting [20,21]. ^177^Lu-labeled carriers were efficiently taken up by T47D breast cancer cells in vitro, outperforming the free ligand and proving the benefit of the delivery vehicle. Upon intratumoral injection, the nanocarrier was retained in the xenograft breast tumor for up to 96 h. The radioisotope allowed not only the effective delivery of radiation in the absorbed dose of 2 Gy per MBq but also convenient optical imaging. Wang and colleagues exploited functionalized hybrid PAMAM dendrimers to target GRPR-positive lung cancer and, likewise, they showed very promising results, including selective absorption in the HEL-299 lung cancer cell line and beneficial retention in tumors in vivo after intratumoral injection [22]. However, gold nanoparticles alone can also serve as promising nanocarriers. Silva et al. used them to design GRPR-targeted imaging probes for the SPECT and MRI imaging of prostate cancer with ^67^Ga and Gd, respectively [23]. Interestingly, they showed that gold nanoparticles, both pristine and labeled with the latter element, can lead to substantial radiosensitization effects upon gamma irradiation. Lastly, liposomes, which are some of the most successful nanostructures, were shown to successfully deliver radioisotopes. The team of Accardo worked with carriers assembled from custom amphiphilic moieties containing a DTPA radioisotope chelator for ^111^In delivery, as well as a (7–14) bombesin fragment for GRPR targeting [24]. Moreover, the hydrophilic compartment of the liposome was used to encapsulate cytotoxic drugs such as doxorubicin. The obtained theranostic radionanocarriers were examined in a prostate cancer model and were shown to selectively bind to malignant cells in vitro and substantially prevent tumor growth in vivo.

Radiation in nanomedicine is useful not only to assure the biological activity of the designed nanosystems; it can be also a very promising tool for various nanoparticles’ synthesis [25,26,27,28,29]. A unique feature of radiation is the fact that the energy required for the initiation of the reactions leading to the formation of nanostructures is physically delivered to the system by a stream of high-energy particles and/or electromagnetic waves. Hence, there is no need for additional initiators, catalysts, or other harmful chemicals that need to be thoroughly removed from the final product upon completed synthesis [30]. This fact renders the obtained products more cost-effective and safer at the same time. An example of a radiation-derived nanostructure is a polymer nanogel—crosslinked polymer networks on the nanometer scale [31,32]. Their outstanding properties, such as biocompatibility, high water content, tailorable size, surface properties, and chemical reactivity, make them perfect candidates for drug delivery applications, and research interest in radiation-synthesized polymer nanogels is prominent [33,34,35]. Among many polymers that may be used to synthesize nanogels, poly(acrylic acid) (PAA) stands out as a particularly promising material. For a general description and characteristics of radiation-synthesized PAA nanogels, the reader is directed to previously published studies [32,36,37,38,39]. Due to the abundant carboxylic groups, PAA is stimuli-responsive (to pH and ionic strength changes) and markedly hydrophilic; it forms structures that are highly colloidally stable as a consequence of electrostatic stabilization [40]. Moreover, it can be relatively easily derivatized with various biologically active moieties, such as peptides [38,41]. 

Despite the great deal of focus on the basic processes leading to the formation of poly(acrylic acid) nanostructures during radiation processes [32,36,37,42], so far, little has been reported on the applications of these promising materials [43,44,45]. In the existing literature, PAA nanogels synthesized with radiation, or any other method, are not broadly studied. In general, most of the applications of poly(acrylic acid) in nanogel science focus on copolymers (with other materials such as poly(N-isopropylacrylamide) [46,47,48,49,50]), interpolymer complexes (e.g., with poly(N-vinylpyrrolidone) [30,51] or poly(ethylene oxide) [52]), and hybrid organic–inorganic structures [41,53,54,55]. Acrylic acid can be also grafted onto other nanoparticles to provide carboxylic groups [29,56], but nanoparticles based solely on PAA are rarely used despite their interesting properties. Therefore, in our group, we explore this field—we have already reported a targeted radionanomedicine system’s synthesis and thorough characterization [38]. 

The targeting of nanosystems to prostate (or any) cancer can be executed in two basic ways: by passive and active strategies. The latter requires the application of a functional ligand able to foster specific accumulation at the site of interest based on the avidity of the receptors overexpressed in the malignancy. Therefore, the choice of the appropriate targeting ligand is a crucial part of the design of such specialized nanosystems for cancer management. Peptides serve this purpose well, as they are less immunogenic, relatively cheap, and can efficiently bind to specific cellular receptors. However, an equally important subject is the method of bringing the targeting moiety and the carrier particle together. One of the most common ways to achieve this is to chemically bind them with a covalent bond. The type of bond strongly depends on the available functional groups and the desired mode of operation of the designed nanosystems—for a carrier with carboxylic groups, such as PAA nanogels and peptide ligands, a convenient solution is the formation of the amide bond. Various conjugation protocols and chemistries can be exploited; however, 1-ethyl-3-(3-dimethylaminopropyl)carbodiimide (EDC) chemistry is probably the most frequently used bioconjugation system and certainly the most popular carbodiimide-based system used in the coupling of carboxylates and amines [57]. Usually, to improve the conjugation yield, EDC is used in combination with N-hydroxysuccinimide or N-hydroxysulfosuccinimide (sulfo-NHS). The major advantage of this reaction is that both EDC and NHS/sulfo-NHS, as well as the reaction byproducts, are water-soluble, so they can be easily removed by dialysis against water or with centrifugal filters; hence, there is no need for extensive purification procedures. This system, however, is not the only available option for conjugation in an aqueous reaction medium. For example, 4-(4,6-dimethoxy-1,3,5-triazin-2-yl)-4-methylmorpholinium chloride (DMTMM) is an emerging coupling reagent in the field of nanosystem functionalization. It originated from bioorganic chemistry, where it was predominantly used for the efficient synthesis of peptide chains. Since its advent [58], some improvements have even been proposed in terms of counterions [59,60], to increase its efficiency in cases of challenging syntheses [38]. These compounds, however, require organic solvents as reaction media; hence, a cost-effective and highly water-soluble chloride remains a noteworthy alternative for EDC/NHS-based procedures, with proven applicability in nanostructure modifications [61,62,63,64,65].

Herein, we describe our efforts to assess poly(acrylic acid) nanogels functionalized with Lys1Lys3-bombesin(1–14) modified with a 1,4,7,10-tetraazacyclododecane-1,4,7,10-tetraacetate (DOTA) chelator, as a carrier for the targeted delivery of Lu-177 and Y-90 radionuclides. To synthesize nanogels of different sizes, we irradiated dilute aqueous solutions of poly(acrylic acid) of varying nominal molecular weights, with short pulses of accelerated electrons from a linear accelerator. This method was developed and thoroughly studied in our laboratory [36,37,42,66]. The functionalization of nanogels of various sizes was systematically investigated to achieve the most beneficial yield, process robustness, and labor effectiveness. Lastly, we tested the in vitro and in vivo performance of the obtained nanoplatform on a prostate cancer model.

## 2. Materials and Methods

### 2.1. Materials

Three batches of linear poly(acrylic acid) (PAA) with nominal average molecular weights of 450 kDa (450PAA; actual value of the weight-average molecular weight determined by static laser light scattering: M_w_ = 494 ± 25 kDa; Cat. No. 03312-100, Lot: 697844; Polysciences Inc., Hirschberg an der Bergstrasse, Germany), 250 kDa (250PAA; actual value determined by static laser light scattering: M_w_ = 114 ± 8 kDa; 35 wt.% solution in H_2_O, Cat. No. 416002-500ML, Lot STBG9347, Sigma Aldrich, Steinheim, Germany), and 30 kDa (30PAA; chain too short for static light scattering measurement; 30 wt.% solution in H_2_O, Cat. No. 24771-250, Lot a823122, Polysciences Inc., Warrington, PA, USA) were used throughout the presented research, without further purification. Perchloric acid (HClO_4_, 70%, Sigma-Aldrich, Poznan, Poland) and 1 M standard solution of sodium hydroxide (NaOH, POCH, Gliwice, Poland) were used for the synthesis and characterization of the nanogels, respectively. The filtration of samples was performed with Minisart NML cellulose acetate syringe filters (Sartorius Stedim Biotech GMBH, Göttingen, Germany). Bombesin derivate peptides, Lys1Lys3-bombesin(1–14) (BN) with sequence KQKLGNQWAVGHLM(NH_2_) and Lys1Lys3(DOTA)-bombesin(1–14) (BD) with sequence KQK(DOTA)LGNQWAVGHLM-(NH_2_), where DOTA stands for 1,4,7,10-tetraazacyclododecane-1,4,7,10-tetraacetate, were supplied by either Lipopharm (Zblewo, Poland) or Biolim (Gdynia, Poland). Sodium perchlorate monohydrate (NaClO_4_·H_2_O), EDC, NHS, DMTMM, 2-(N-morpholino)ethanesulfonic acid (MES), N-[tris(hydroxymethyl)methyl]-2-aminoethanesulfonic acid (TES), cellulose dialysis tubing of typical molecular weight cut-off 14 kDa, and Amicon^®^ Ultra Centrifugal Filter Units of typical cut-off of 30 kDa and various volumes (0.5 mL to 15 mL) were provided by Sigma-Aldrich (Poznan, Poland). The Novagen^®^ bicinchoninic acid protein assay kit (BCA assay) was provided by Sigma-Aldrich, Poznan, Poland. TKA micropure filtered water (conductivity 0.05 µS cm^−1^, 0.2 µm filter, Thermo Fisher Scientific, Waltham, MA, USA) was used throughout all the experiments. 

The following chemicals were used for radiolabeling: L(+)-ascorbic acid (Cat. No. 141013.121, PanReac AppliChem, Darmstadt, Germany), sodium hydroxide 30% solution (NaOH, 99%, Merck, Darmstadt, Germany), diethylenetriaminepentaacetic acid (DTPA, Merck, Darmstadt, Germany). Lutetium-177 (LutaPol) as lutetium chloride of specific activity higher than 555 MBq/mg Lu in 0.04 N HCl and yttrium-90 (ItraPol) as yttrium chloride in a 0.04–0.05 N HCl of 0.925–37 GBq in a volume 0.010–2 mL were produced at the Radioisotope Centre POLATOM (Otwock, Poland).

### 2.2. Electron Beam Dosimetry

Electron beam dosimetry was performed using alanine pellets from Bruker (Billerica, MA, USA), and readings of absorbed radiation doses were acquired using an e-scan electron paramagnetic resonance spectrometer (Bruker, Billerica, MA, USA).

### 2.3. Synthesis and Characterization of PAA Nanogels

PAA nanogels (NG), both based on 250PAA (250NG) and 450PAA (450NG), were synthesized as previously described [37]. Briefly, PAA solutions acidified to pH 2.0 with HClO_4_ were subjected to a series of short pulses (4 µs; 0.5 Hz) of accelerated electrons (electron energy 6 MeV) from a linear accelerator (ELU-6, Elektronika, Russia) in a preparative pulsed radiolysis regime, using a closed-loop system. Concentrations of stock solutions for particular nanogel samples and total absorbed doses of radiation are specified in Table 1. Concentrations and doses (as a derivative of required irradiation cycles and the dose per pulse) were chosen based on a previously performed systematic analysis (Supplementary information, Additional File S1).

The obtained nanogels were used after dialysis, which allowed the removal of the unwanted perchloric acid and any potential low-molecular-weight byproducts of the reaction. Briefly, upon completed irradiation, stock solutions were brought to pH 7.0 with 1 M NaOH. After at least an hour of equilibration, samples were moved to cellulose dialysis tubing and dialyzed against water. After completed dialysis (up to 7 days), samples were filtered with a set of acetate cellulose syringe filters with a final pore size of 0.2 µm for sample preservation during storage. Filtered samples were aliquoted in sterile 50 mL conical tubes for further use. The size of the nanocarriers was measured using dynamic light scattering (DLS) with a ZetaSizer Nano (Malvern Instruments, Malvern, UK) and expressed as the hydrodynamic diameter (D_h_). A study on the size of nanogels upon completion of the purification process was performed in an aqueous solution at 22 °C (room temperature) in a thermostated device.

Separate aliquots of irradiated solutions (not subjected to purification procedures) were used for the characterization of the products using static light scattering (SLS). Briefly, samples were supplemented with NaClO_4_ to a final concentration of 0.5 M and the pH was brought to 10.0 using 1 M NaOH. SLS data were acquired using a BI-200SM goniometer (Brookhaven Instruments Corporation, Holtsville, NY, USA) with an Innova 90 C green Ar ion laser (λ = 514.5 nm; Coherent, Santa Clara, CA, USA) at 25.0 ± 0.1 °C. The Zimm algorithm was used for data analysis [67], with dn/dc = 0.30 cm^3^/g [68], to yield weight-average molecular weights of PAA macromolecules and nanogels. Prior to SLS measurements, samples were filtered through 0.45 µm acetate cellulose syringe filters.

### 2.4. Synthesis and Characterization of Nanocarriers 

**Figure 1:**
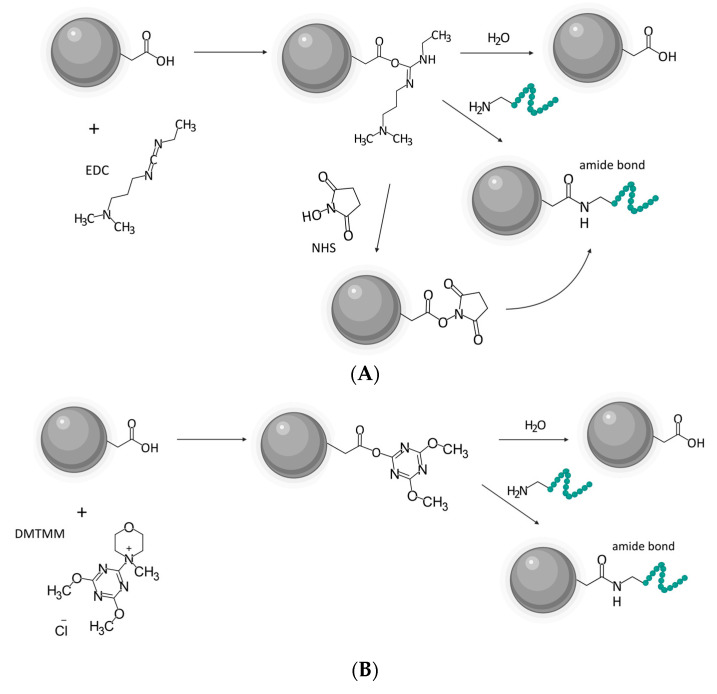
(**A**) Reaction scheme of the conjugation based on EDC/NHS. (**B**) Reaction scheme of the conjugation based on DMTMM. Both figures created with BioRender.com, chemical structures rendered with MarvinSketch.

Upon completed irradiation, purification, and characterization, the nanogels were further processed towards targeted nanocarriers. The method for the nanogels’ functionalization was adopted from Nagórniewicz et al. [69], with some modifications. The conjugation of BN to 250NG nanogels (30 peptides per nanoparticle) was performed with two distinct coupling chemistries, namely carbodiimide with EDC/NHS [57] (Figure 1A) and triazine with DMTMM [58] (Figure 1B).

The condensation of the primary amines in the peptides with carboxylic groups present in PAA was performed in varying environments, and the following variables were altered: reaction scheme, –COOH activation chemistry, and pH. Once the final protocol for Lys1Lys3-bombesin(1–14)-functionalized 250NG (250NGBN) was established, the effectiveness of the chosen process was confirmed with BD (250NGBD), as well as on

- 450NG nanogels at 170 BN and BD peptides per nanoparticle (450NGBN and 450NGBD, respectively);
- 30PAA molecules at 4 BN and BD peptides per chain (30PAABN and 30PAABD, respectively).

The density of peptide grafting along the PAA main chain is the result of a compromise. On the one hand, more peptide–DOTA moieties mean higher specific activity of the radioisotope and possibly also the higher affinity of the nanogel particles to the target cells. On the other hand, obviously, there is a spatial limit—for steric reasons, the relatively large bombesin–DOTA moieties cannot be grafted on the main chain at overly low distances from each other. Furthermore, an overly high peptide/acrylic acid ratio could reduce the effective negative charge of the product, thus impairing its colloidal stability. In our case, based on our earlier experience with PAA–bombesin–DOTA nanogels [38], we aimed at having approximately one peptide grafted per 100 acrylic acid monomer units (this corresponds to the weight ratio of peptide/PAA of ca. 1/3.5). This density was expected to be high enough to provide efficient uptake by the tumor cells and was still synthetically achievable without leaving activated carboxylate groups unoccupied. Moreover, the resulting nanoconstructs were expected to retain the excellent colloidal stability of the parent PAA nanogels.

#### 2.4.1. Multistep Reaction with EDC/NHS

The first attempts at NGBN synthesis were based on the EDC/NHS chemistry, according to Nagórniewicz et al. [69]. The multistep nature of the protocols stems from the fact that the reactions in the EDC/NHS methodology are very sensitive to the pH. The best pH for –COOH activation is acidic, even as low as 3.5–4.5, but EDC is very unstable in such acidic conditions. Moreover, the intermediate formed upon activation on the –COOH group reacts only with a deprotonated amine; hence, at a low pH, the amine might not be reactive [70]. Therefore, it was considered beneficial to change the pH between the activation and conjugation steps. 

Then, 400 µL of nanogel aqueous solution (0.72 mg/mL) was mixed with 2 µmol EDC and 6.7 µmol NHS dissolved in 100 µL of 5× concentrated appropriate buffer, which was used to maintain the pH of the reaction. After completing the 90 min activation of the carboxylic groups, samples were subjected to buffer exchange by triple washing with an appropriate buffer using Amicon^®^ centrifugal filters. Samples were retrieved from filters by means of pipetting. Recovered samples in the buffer with the desired pH (at least ca. 400 µL) were supplemented with BN (1 mg/mL) to the final amount of 30 peptides per nanoparticle and reacted overnight under gentle agitation for conjugation to occur. Finally, samples were purified with Amicon^®^ filters by triple washing with PBS, recovered from filters, and stored at 4 °C until further experiments. The detailed conditions of the particular reaction schemes are specified in Table 2.

#### 2.4.2. One-Pot Reaction with EDC/NHS 

To check if the washing step influenced the reaction yield, a one-pot reaction using EDC/NHS was attempted. First, 400 µL of nanogel aqueous solution (0.72 mg/mL) was mixed with 2 µmol EDC and 6.7 µmol NHS dissolved in 100 µL of 5× concentrated appropriate buffer, which was used to maintain the pH of the reaction. After 90 min of activation, BN (1 mg/mL) was added, and the reaction was carried out overnight under gentle agitation for conjugation to occur. Finally, samples were purified with Amicon^®^ filters by triple washing with PBS, recovered from filters, and stored at 4 °C until further experiments. The detailed conditions of the particular reaction schemes are specified in Table 3.

#### 2.4.3. One-Pot Reaction with DMTMM

To investigate whether a change in the activating agent would improve the reaction yield, we used DMTMM to activate –COOH groups [70]. DMTMM works similarly to EDC—it activates the carboxylic group, and the active intermediate is able to react with the amine available in BN, by nucleophilic substitution, to form a stable amide bond [71]. First, 400 µL of nanogel aqueous solution (0.72 mg/mL) was mixed with 2 µmol DMTMM dissolved in 100 µL of 5× concentrated appropriate buffer, which was used to maintain the pH of the reaction. After 90 min of activation, BN (1 mg/mL) was added, and the reaction was carried out overnight under gentle agitation for conjugation to occur. Finally, samples were purified with Amicon^®^ filters by triple washing with PBS, recovered from filters, and stored at 4 °C until further experiments. The detailed conditions of the particular reaction schemes are specified in Table 4.

### 2.5. Characterization of Nanocarriers

According to the manufacturer’s instructions, the efficiency of peptide coupling to the nanogels was determined with a micro-scale Novagen^®^ bicinchoninic acid protein assay. Briefly, 25 µL of each sample was pipetted in duplicate into individual wells of a 96-well plate and 200 µL of BCA working reagent was added to each well. Samples were mixed on a plate shaker for 1 min and incubated for 15 min at 60 °C. After 10 min of cooling, the plate was read at the plate reader at 550 nm, and the peptide concentration was calculated based on the standard curve prepared from the proper peptide standard samples (25–500 µg/mL) incubated with BCA working agent on the same plate. The reaction yield was calculated as the % of the peptide mass added to the reaction mix.

The size of the nanocarriers for the colloidal stability study was measured using DLS with a ZetaSizer Nano (Malvern Instruments, Malvern, UK) and expressed as the hydrodynamic diameter (D_h_). A study of colloidal stability in biologically relevant media was performed at 37 °C in a thermostated device; samples were aseptically diluted in appropriate media, tightly sealed, and stored in a dark incubator at 37 °C for 7 days. Measurements were performed at predetermined time points, as specified in the Results and Discussion section.

### 2.6. Radiolabeling

The samples of NG or PAA coupled with BD were labeled with ^177^LuCl_3_ /^90^YCl_3_ (0.2–0.6 GBq). Then, 0.1 mL of NGBD/PAABD was mixed with 0.2 mL of ascorbic acid buffer (AAB) (50 mg/mL) of pH 4.5–5.0, and 2–20 µL of radionuclide was added. The samples were incubated at 95 ± 5 °C for 15 min.

The final formulation’s radiochemical purity (RCP) was determined by thin-layer chromatography on glass-fiber silica gel-coated plates (ITLC SG) with methanol: 1 M ammonium acetate solution (1:1 *v*/*v*) as a mobile phase. The radiolabeling purity was evaluated in a competitor’s presence (10 mM DTPA) in excess (1:1 *v*/*v*), complexing the unbound or weakly bound (by carboxylic groups of PAA) radionuclide. A sample of 5 µL of radiolabeled NGBD/PAABD was spotted on the strip at a distance of 1 cm from its bottom. To determine the amount of free ^177^Lu/^90^Y- (Rf = 1), [^177^Lu]Lu/[^90^Y]Y-BD (Rf = 0.6) and [^177^Lu]Lu/[^90^Y]Y-NGBD/[^177^Lu]Lu/[^90^Y]Y-PAABD (Rf = 0) strips were developed, and the distribution of radioactivity was determined using a Cyclone^®^Plus (PerkinElmer, Waltham, MA USA) scanner.

### 2.7. Biological Studies

#### 2.7.1. Cell Culture

The human prostate adenocarcinoma cell lines LNCaP and PC-3, acquired from the European Collection of Authenticated Cell Cultures (ECACC) (Sigma Aldrich, Saint Louis, MO, USA), were grown in RPMI 1640 medium (Thermo Fisher Scientific Inc, Waltham, MA, USA). The human prostate adenocarcinoma DU-145 cell line was acquired from the American Type Culture Collection (ATCC) (Manassas, VA, USA) and cultured in DMEM medium (Thermo Fisher Scientific Inc, Waltham, MA, USA). All media were supplemented with 10% heat-inactivated fetal bovine serum (FBS, Thermo Fisher Scientific Inc., Waltham, MA, USA) and 1% penicillin/streptomycin (10,000 U/mL, Thermo Fisher Scientific Inc., Waltham, MA, USA), HEPES buffer, sodium pyruvate, and L-glutamine (Thermo Fisher Scientific Inc., Waltham, MA, USA). Cultures were maintained at 37 °C in a humidified 5% CO_2_ atmosphere. The cells were harvested using trypsin–EDTA solution (0.25% trypsin, 0.02% EDTA, Thermo Fisher Scientific Inc., Waltham, MA, USA) after reaching 80% confluence for further culture or experiments.

#### 2.7.2. Real-Time Quantitative Polymerase Chain Reaction (RTqPCR)

Cells were seeded on 60 mm Petri dishes at the density of 1 × 10^6^ and cultured to reach 90% confluence. After this, the cell medium was removed and cells were harvested using TRIzol™ reagent (Thermo Fisher Scientific Inc., Waltham, MA, USA). Total RNA was isolated using chloroform extraction with subsequent ethanol precipitation. The final product was diluted in 50 µL of sterile deionized water free from RNAses and DNAses (DEPC) and the RNA concentration was determined using a BioDrop DUO spectrophotometer (BioDrop, Cambridge, UK). cDNA was synthesized from 5 µg RNA with an ImProm RT-IITM reverse transcriptase kit (Promega, Madison, WI, USA) according to the manufacturer’s instructions. The relative expression of the *GRPR* gene was analyzed using the LightCycler 96 (Roche, Basel, Switzerland) with ribosomal protein S17 (*RPS17*), ribosomal protein P0 (*RPLP0*), and histone H3.3A (*H3F3A*) as reference genes and Human Reference RNA (Stratagene, San Diego, CA, USA) as a calibrator sample. Data were analyzed according to the ΔΔCt methodology. RTqPCR primers, as listed in Table 5, were designed using the Primer-BLAST software (National Institutes of Health, NIH, Bethesda, MD, USA; https://www.ncbi.nlm.nih.gov/tools/primer-blast/, accessed on 15 June 2020). The melting curve was analyzed for each reaction to confirm the specificity of the product.

#### 2.7.3. Western Blotting

For total protein isolation, cells were plated on 100 mm Petri dishes at the density of 2 × 10^6^ and cultured to reach 90% confluence. Subsequently, cells were mechanically detached using a sterile cell scraper and cell lysates were prepared in RIPA buffer supplemented with 1 mM phenylmethylsulfonyl fluoride (PMSF), as well as a protease and phosphatase inhibitor cocktail (Sigma Aldrich, Sigma Aldrich, Saint Louis, MO, USA). The protein concentration was analyzed with the DirectDetect system (Merck Millipore, Burlington, MA, USA). A sample volume equivalent to 30 mg of protein was mixed with 5 µL Laemmli buffer (bromophenol blue, 8–10% SDS, 20% 2-mercaptoethanol, 40% glycerol, in 0.125 M Tris–HCl) and heated at 95 °C for 5 min. Next, samples were subjected to sodium dodecyl sulfate–polyacrylamide gel (SDS-PAGE) electrophoresis, following wet transfer and non-specific binding site blocking, as previously described [72]. The membrane was incubated overnight with a primary GRPR antibody, as specified in Table 6, in 1% non-fat powdered milk in TBST buffer. The next day, following TBST rinsing (3 × 5 min), the membrane was incubated for 4 h with a solution of phosphatase-conjugated secondary antibody (Sigma Aldrich, Saint Louis, MO, USA), according to the manufacturer’s instructions. After washing, bands were visualized using Novex^®^ AP Chromogenic Substrate (BCIP⁄NBT) (Thermo Fisher Scientific Inc., Waltham, MA, USA). Glyceraldehyde-3-phosphate dehydrogenase (GAPDH) (Santa Cruz Biotechnology, Dallas, TX, USA) was used as a normalization protein. Densitometric analysis of the protein was executed with the ImageJ software, v.1.53i (National Institutes of Health, Bethesda, MD, USA; http://rsb.info.nih.gov/ij/, accessed on 31 March 2021).

#### 2.7.4. Cellular Uptake

To determine the cell internalization of [^90^Y]Y-NGBD, PC-3 cells were plated on 12-well plates (1.5 × 10^6^ cells/well) and incubated in un-supplemented RPMI 1640 medium with the amount of ^90^Y radiolabeled nanocarriers equivalent to 7 pmol of BD or the corresponding activity of ^90^Y at 37 °C for 1 h and 4 h. After the appropriate time, the medium was removed, and cells were washed twice using phosphate-buffered saline (PBS, IITD PAN Wroclaw, Poland). The cells were treated twice with 1 mL of 50 mM glycine in 0.1 M NaCl at a pH of 2.8 and incubated for 5 min at room temperature to collect extracellularly bound compounds. The internalization was determined by measuring the radioactivity of 1 mL of 1 M NaOH used for cell lysis. Total uptake was calculated as the sum of the membrane-bound and internalized fractions measured using the Wallac Wizard 1470 γ counter (PerkinElmer, Waltham, MA USA). As a 100% standard, radiolabeled 250NGBD, 450NGBD, BD, and ^90^Y were used.

### 2.8. In Vivo Studies

BALB/c NUDE male mice (BALB/c AnN-*Foxn1*^nu/nu^/Rj, 6–7 weeks old, mean body mass of 25 g) were purchased from JanvierLabs (Le Genest-Saint-Isle, France). BALB/c mice (5–7 weeks old, mean body mass of 24 g) were purchased from the M. Mossakowski Institute of Experimental and Clinical Medicine, Polish Academy of Sciences in Warsaw (Poland). On arrival, animals were housed for 7 days in groups of five in standard cages (BALB/c mice) or IVS cages (BALB/c NUDE) in the animal facility of the Radioisotope Centre POLATOM (Otwock, Poland). They were housed in a quiet room under constant conditions (22 ± 2 °C, 50% relative humidity, 12-h light/dark cycle with dark period from 7 p.m. to 7 a.m.) with free access to standard food and water. Veterinarian staff and investigators observed the mice daily to ensure animal welfare and determine if humane endpoints were reached (e.g., hunched and ruffled appearance, apathy, ulceration, severe weight loss, tumor burden).

An experimental tumor murine model was induced using PC-3 cells, which grew to 80–90% confluence before trypsinization and formulation in Matrigel™ Basement Membrane Matrix (Corning, Bedford, MA, USA) for implantation into mice. The mice were subcutaneously (s.c.) injected in the shoulder with 200 µL bolus containing a suspension of 3 × 10^6^ freshly harvested cell line PC-3 in Matrigel™. This procedure was performed under anesthesia with 2% isoflurane. The animals were kept under pathogen-free conditions, and experiments were performed 1–2 weeks later when tumors had reached a volume of approximately 150 ± 60 mm^3^.

**Figure 2:**
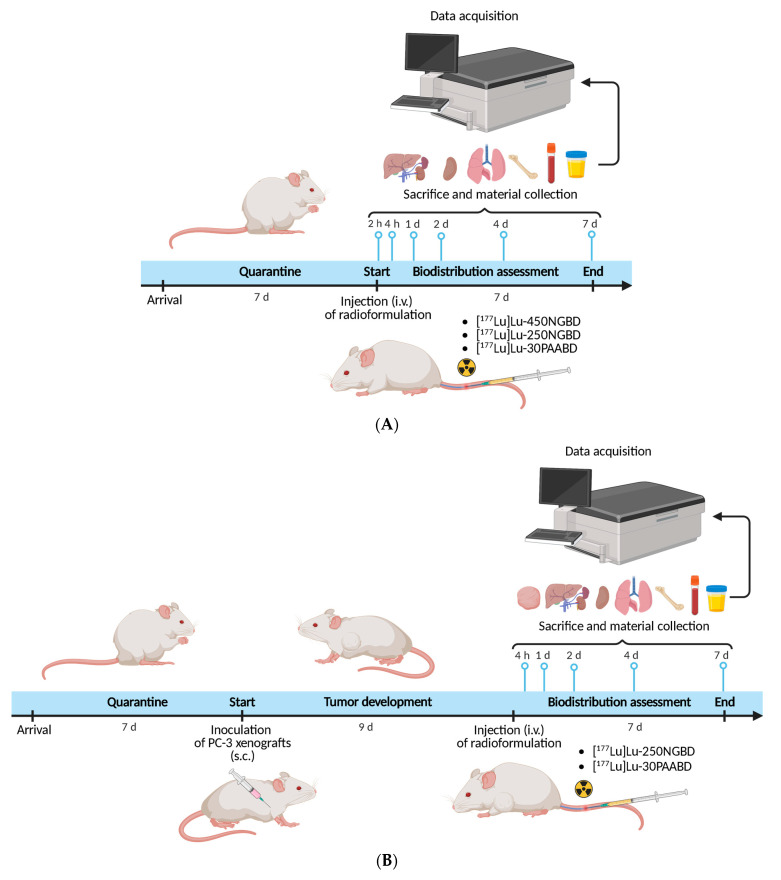
Experimental timeline schemes for in vivo experiments with targeted nanocarriers of biologically active radioisotopes. (**A**) Assessment of the physiological biodistribution of nanocarriers in healthy BALB/C mice. (**B**) Assessment of the physiological biodistribution of nanocarriers in tumor-bearing BALB/C NUDE mice. Created with BioRender.com.

The standard protocol involved animals (healthy and xenografted) randomized into fixed groups (five mice per group). Before injection, the labeled compounds were diluted in 0.9% NaCl and then intravenously injected (0.1 mL per mouse). At established time points after injection, the animals were euthanized by cervical dislocation and dissected. Selected organs and tissues were weighed, and their radioactivity was measured in a gamma counter equipped with a NaI(Tl) crystal. The physiological distribution was calculated and expressed in terms of the percentage of administrated dose found in each of the selected organs or tissues per gram (%ID/g) with suitable standards of the injected dose. Specific timelines for both protocols are depicted in Figure 2.

The physiological distribution of three radioformulations, [^177^Lu]Lu-450NGBD, [^177^Lu]Lu-250NGBD, and [^177^Lu]Lu-30PAABD, in healthy BALB/c mice was performed at five or six time points (2 h, 4 h, 1 day, 2 days, 4 days, and 7 days post i.v. injection) (Figure 2A). For the experiment, mice were injected with a dose of 1.5 nmol BD in 0.1 mL and activity of 7 MBq.

The physiological distribution in xenografted mice of two radioformulations, [^177^Lu]Lu-250NGBD and [^177^Lu]Lu-30PAABD, was performed at five time points (4 h, 1 day, 2 days, 4 days, and 7 days post i.v. injection) (Figure 2B). For the experiment, mice were injected with a dose of 1.3 nmol BD (8 MBq) in 0.1 mL and 2.2 nmol BD (7.8 MBq) in 0.1 mL of [^177^Lu]Lu-250NGBD and [^177^Lu]Lu-30PAABD, respectively.

### 2.9. Statistical Analysis

Results are expressed as mean ± SEM. Statistical analysis was performed with one-way analysis of variance (Section 3.3.3.) and two-way analysis of variance (Section 3.3.2.) using the GraphPad Prism 10.0 software (GraphPad Software, La Jolla, CA, USA). *p* < 0.05 was considered statistically significant. 

The Spearman’s rank correlation coefficient was used to determine the strength and direction of correlation between the time after the intravenous injection of [^177^Lu]Lu-30PAABD/[^177^Lu]Lu-250NGBD and its radioactivity uptake in blood as %ID/g.

## 3. Results and Discussion

Prostate cancer is the most prevalent cancer in males, increasingly diagnosed in developed countries. We have investigated the application of radiation-synthesized PAA nanogels in the theranostic management of prostate cancer, namely the targeted delivery of theranostic isotopes. We have studied the complete process, from the synthesis of nanogels by irradiation of the dilute polymer solutions (changes in the weight-average molecular weight, radius of gyration, and coil density as a function of absorbed dose, Figures S1–S3, indicating formation of nanogels), through functionalization, radiolabeling, and in-vitro assessment, to in vivo biodistribution, pharmacokinetics, and theranostic potential in tumor-bearing mice. To the best of our knowledge, this is the first complete report on a poly(acrylic acid)-based nanocarrier system for radioisotopes.

Functional nanosystems based on poly(acrylic acid) were hereby synthesized from three types of carriers, including nanogels based on two batches of PAA with different nominal average molecular weights (250 kDa, 450 kDa) and linear PAA with a nominal average molecular weight of 30 kDa as a control for in vivo pharmacokinetics. Nanogels were synthesized according to two chosen protocols established upon the systematic investigation of the synthesis (Additional File S1). The molecular weights as well as the hydrodynamic diameters of the nanogels are specified in Table 7. 

### 3.1. Nanocarrier Functionalization

**Figure 3:**
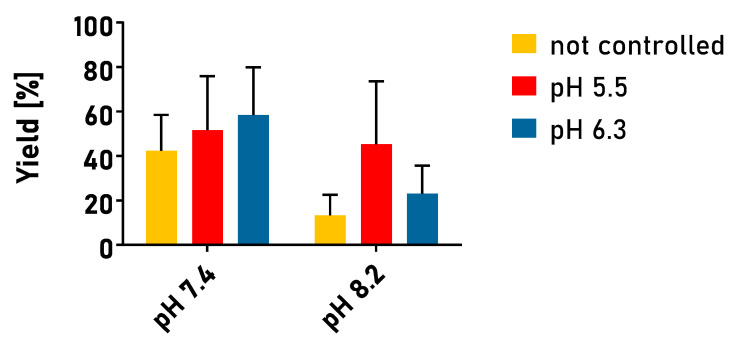
Conjugation yields were obtained in the multistep process at varying pH. pH 5.5 and 6.3 were maintained using 0.1 M MES buffer; pH 7.4 was maintained using PBS buffer; pH 8.2 was maintained using 0.1 M TES buffer. In the non-controlled process, conjugating agents were diluted in demineralized water. Colors indicate pH maintained during the activation step of the reaction; X-axis indicates pH maintained in the conjugation phase. Reactions performed in triplicate. Results are expressed as mean yield ± SEM.

The conjugation yields obtained in a multistep process performed at a varying pH are summarized in Figure 3. It was found that, as expected, the varying pH influenced the yield of the process. Moreover, the multistep process also led to relatively high variability in the obtained, results as expressed by the high error bars. In general, it was found that a higher pH during the conjugation step did not necessarily benefit the reaction with higher conjugation efficiency. The yields of the reactions for conjugation at pH 8.2 were substantially worse than at pH 7.2. For the reaction performed without any pH control during the activation step, approximately 42% and 13% yields were obtained for conjugation performed at pH 7.4 and 8.2, respectively. pH control improved the reaction efficiency; however, in none of the examined protocols did the yield exceed 60%. One of the possible reasons for this result is that the washing step, which takes time and manipulation, quenches the active sites. The obtained results prompted us to examine other conjugation protocols. 

**Figure 4:**
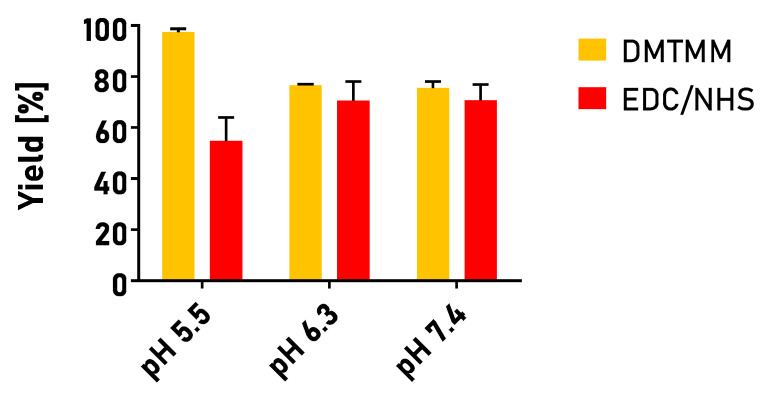
Conjugation yields obtained in the one-pot process at varying pH, using various conjugation chemistries. pH 5.5 and 6.3 were maintained using 0.1 M MES buffer; pH 7.4 was maintained using PBS buffer. Reactions performed in triplicate. Results are expressed as mean yield ± SEM.

To investigate whether the washing step influenced the yield of the reaction and to simplify the process at the same time, we decided to omit the buffer exchange and maintain the pH at the same level throughout the process. Such a one-pot procedure was examined not only with EDC/NHS but also with an emerging conjugating agent, DMTMM, which is not frequently used but is also water-soluble and has been proven efficient for various nanosystem functionalization procedures [64,73,74]. The conjugation yields obtained in the one-pot processes performed at a varying pH are summarized in Figure 4.

The simplification of the process led to a notable increase in the reaction yield for the EDC/NHS-mediated syntheses, particularly at higher pH values. The process efficiency was improved to 54% for a pH of 5.5 and to 71% for pH 6.3 and 7.4, respectively. The obtained results prove the disadvantageous influence of buffer exchange via washing on the process yield. Nevertheless, it was found that changing the –COOH activating agent led to an even greater improvement in the reaction yield. At pH 5.5, more than 97% conjugation efficiency was obtained; therefore, the one-pot protocol exploiting DMTMM and pH 5.5 maintained with MES buffer was chosen for the synthesis of targeted nanocarriers for further studies. 

**Figure 5:**
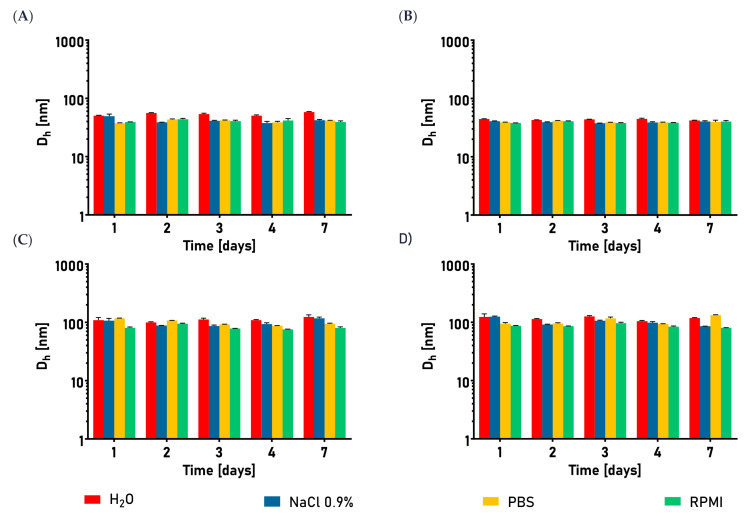
Hydrodynamic sizes of nanogels and corresponding nanocarriers upon functionalization with bombesin, in various biologically relevant media, at 37 °C, as a function of storage time. (**A**) 250NG. (**B**) 250NGB. (**C**) 450NG. (**D**) 450NGB. Results are expressed as mean hydrodynamic diameter ± SEM.

Once the conjugation protocol was established, multiple batches of the nanocarriers were produced and used for further experiments. In order to check if the obtained products were colloidally stable, a systematic investigation of the nanocarriers’ hydrodynamic diameters was performed in various media, including those biologically relevant, considering the planned in vitro experiments. As shown in Figure 5, there was no significant change in the size of the nanocarriers at chosen densities of peptide grafting, in the period of at least 7 days after conjugation, which was sufficient time to perform all the required tests with a single production batch. However, since functionalization did not substantially influence the nanogels’ size or stability, based on our previous research, we assumed that the samples would be colloidally stable at least for one month [39].

### 3.2. Radiolabeling

The radiolabeling study revealed that PAA nanogels coupled with BD could be effectively labeled with lutetium-177 and yttrium-90, and both radioconjugates were stable in radiolabeling buffer (AAB) and human serum (HS) for up to two weeks. The summarized results of the study are presented in Table 8 and Table 9. After radiolabeling, the 30PAABD had to be purified due to the labeling yield. The radiochemical purity (RPC) of 30PAABD decreased from 96.1 ± 1.72 to 93.5 ± 1.21 and from 94.5 ± 1.36 to 93.2 ± 0.26 within 1 day for ^177^Lu and ^90^Y, respectively. [^177^Lu]Lu/[^90^Y]Y-30PAABD was purified on Amicon^®^ Ultra Centrifugal Filter Units of a typical cut-off of 10 kDa. The sample of 0.3 mL was centrifuged at 13,500 cpm for 30 min and washed by the two-time addition of 0.5 mL AAB followed by centrifugation. The purified radiolabeled BD was collected from the fraction before the filtration membrane.

### 3.3. Targeted Delivery of Radiolabeled Nanocarriers

#### 3.3.1. Gastrin-Releasing Peptide Receptor Expression

**Figure 6:**
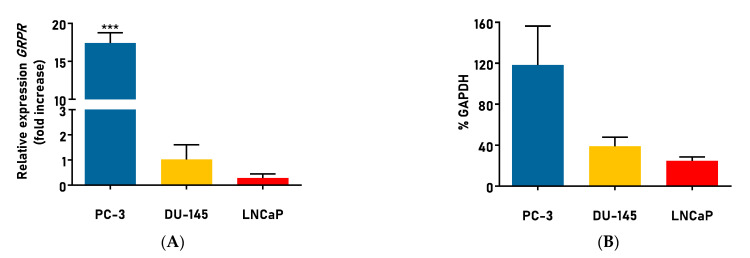
Expression of gastrin-releasing peptide receptor in prostate cell lines. (**A**) GRPR gene expression. Results are expressed as mean fold increase ± SEM. *p* < 0.05 is considered statistically significant, *** *p* < 0.001. (**B**) GRPR protein expression. Results are expressed as mean fold increase ± SEM.

In order to choose the appropriate cell lines for further studies and confirm their GRPR receptor expression, as a first step, we screened three adenocarcinoma cell lines by means of transcript and proteome analysis. According to current knowledge, the prostate cancer cell lines that express GRPR are predominantly the PC-3, DU-145, and LNCaP cell lines. We found that the highest expression of GRPR, both on the mRNA level and the protein level, was detectable in the PC-3 cell line (Figure 6), which was in agreement with the current state of knowledge [75].

#### 3.3.2. Nanocarrier Uptake in GRPR-Positive Cell Line

**Figure 7:**
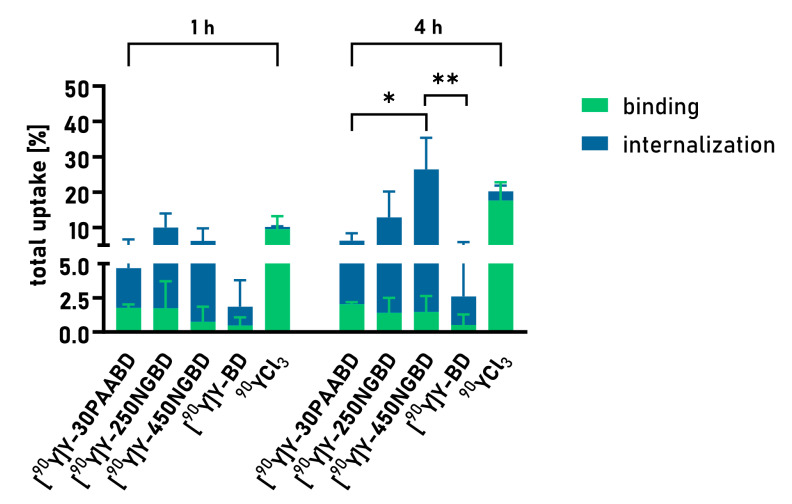
Total uptake of radioactivity upon treatment of PC-3 cells with ^90^Y in various forms and at different times. As a 100% standard, radiolabeled 30PAABD, 250NGBD, 450NGBD, and BD as well as ^90^Y were used. Results are expressed as stacked mean surface binding and internalization ± SEM. *p* < 0.05 is considered statistically significant, * *p* < 0.05, ** *p* < 0.005.

The targeted delivery of radioisotopes is assumed to enhance the radioactivity uptake in cancer cells. In order to investigate this, the total binding (sum of internalization and surface receptor binding) of the targeted nanocarriers 30PAABD, 250NGBD, and 450NGBD was determined using the PC-3 cell line (Figure 7). It was found that the chosen densities of peptide grafting allowed the uptake of the nanosystems, and the total uptake of the radiation gradually grew over time. Two-way analysis of variance revealed that the effect of the type of the sample on the total uptake value was statistically significant (*p* < 0.001 for binding and *p* < 0.005 for internalization). Moreover, the internalization of radioactivity was substantially increased upon application of the delivery vehicle, in comparison to the free radioisotope and carrier-free radiolabeled BD peptide. In the in vitro setting, 450NGBD provided the greatest internalization of almost 27% of radioactivity after 4 h of incubation. This might be connected with the fact that 450NGBD carries the largest number of radionuclides per nanoparticle, so, with a similar number of internalization events, this carrier would direct more radioactivity into the cell in comparison to constructs carrying a smaller number of radionuclides.

**Figure 8:**
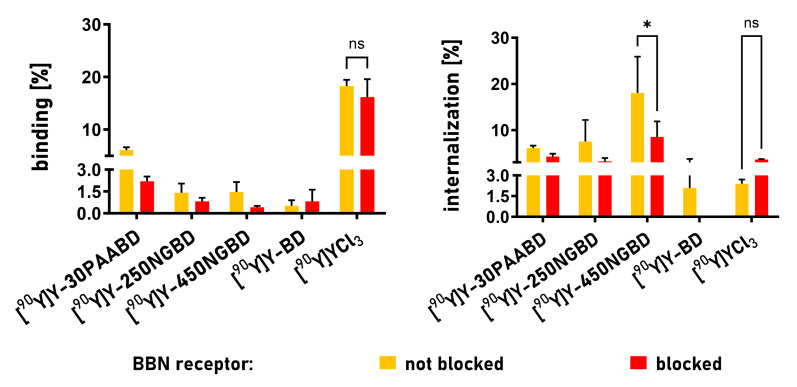
Surface receptor binding and internalization of radioactivity upon treatment of PC-3 cells with ^90^Y in various forms for 4 h. Results are expressed as mean ± SEM of receptor binding and internalization, respectively. *p* < 0.05 is considered statistically significant, * *p <* 0.05, ns—not significant.

In order to confirm that the uptake was connected with the presence of the GRPR in the cells, we used an excess of non-radiolabeled BN to block the receptor. Upon saturating the receptors with 0.7 nmol of the peptide, specific internalization was greatly inhibited for the systems where the radioisotope was delivered using BD-targeted carriers: nanogels and linear PAA (Figure 8).

#### 3.3.3. In Vivo

**Figure 9:**
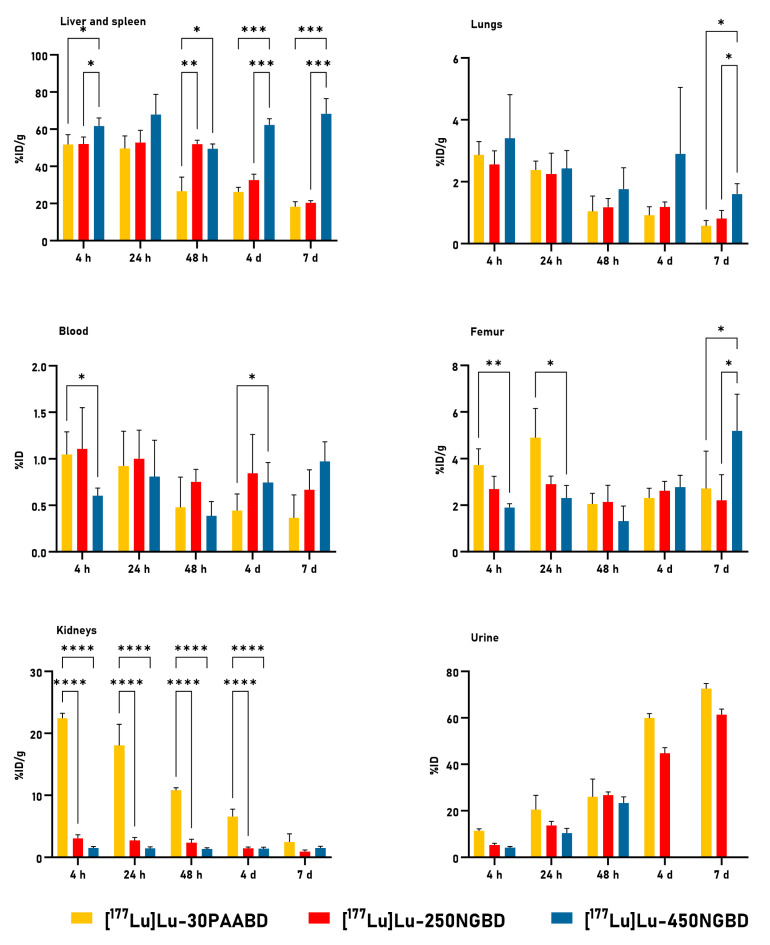
The uptake of [^177^Lu]Lu-PAA/[^177^Lu]Lu-NG in selected organs (%ID/g) and urinary elimination (%ID) in healthy mice. Results are expressed as mean ± SEM. *p* < 0.05 is considered statistically significant, * *p* < 0.05, ** *p* < 0.005, *** *p* < 0,0005, **** *p* < 0.0001. %ID—percentage of injected dose; %ID/g—percentage of injected dose per gram of selected organ or tissue.

The 250NGBD and 450NGBD followed a similar distribution in the murine model (healthy mice). As much as 50% ID/g of both [^177^Lu]Lu-450NGBD and [^177^Lu]Lu-250NGBD accumulated in the liver and the spleen in a time-dependent manner. By contrast, all other organs examined retained only small amounts of the NPs, at levels below 5%. Full data (tissue distribution) are listed in Additional File S2: Tables S1–S3. The accumulation of nanocarriers in critical organs, i.e., the liver, spleen, femur, lungs, and kidneys, as well as in blood and urinary elimination, are presented in Figure 9.

Statistically significant accumulation differences for all NPs in the liver and the spleen were noted, except at a 24 h time point after administration. Increased liver and spleen uptake was noted after 48 h for [^177^Lu]Lu-450NGBD. The above observation and data from Tables S1–S3 point to the liver as the main organ that metabolizes and removes nanoparticles from the body. Due to the long metabolism time of radionanocarriers, their bioavailability is very high. The high uptake of [^177^Lu]Lu-450NGBD in the liver may have led to radiation-induced toxicity with the development of numerous symptoms and chronic side effects, including late fibrosis, DNA damage, and reactive oxygen species (ROS) [76,77]. However, during macroscopic observation, we found no changes in the liver, and histopathological tests were not performed.

The literature data on the biodistribution of numerous nanoplatforms studied [78] reveals that nanoparticles of almost all sizes are mainly accumulated in organs such as the liver, spleen, and lungs. In our study, the highest uptake in the lungs was below 4% ID/g ([^177^Lu]Lu-450NGBD at 4 h) and decreased over time to a value ca. 1% ID/g for [^177^Lu]Lu-30PAABD and [^177^Lu]Lu-250NGBD and below 2% ID/g for [^177^Lu]Lu-450NGBD.

The 30PAABD was more quickly taken up by the kidneys and eliminated from the body than 250NGBD and 450NGBD (22.4 ± 0.80% ID/g, 3.1 ± 0.57% ID/g and 1.5 ± 0.23% ID/g at 4 h, respectively).

Based on the Spearman’s rank correlation results shown in Table 10, for all nanocarriers, we found a negative association between the variables: time after intravenous injection of [^177^Lu]Lu-30PAABD/[^177^Lu]Lu-250NGBD and its radioactivity uptake in blood as %ID/g). However, the coefficients r_s_ = −0.9276 and r_s_ = −1.000 for nanocarriers based on 250PAA and 30PAA, respectively, indicate a negative monotonic relationship that is statistically significant. A weak relationship was observed for nanocarriers based on 450NG, but, in this case, it was a negative relationship, r_s_ = −0.1429, *p* = 0.8028. For 450NGBD, the %ID/g of radioactivity uptake in the blood was not associated with the time post i.v. injection (*p* > 0.05), but, for 30PAABD and 250NGBD, a longer time post i.v. injection tends to be associated with lower radioactivity uptake values in blood.

Based on the results from an in vivo study with healthy mice, only two nanocarriers were chosen for the in vivo study in subcutaneous tumor models. These were 30PAABD and 250NGBD due to their favorable distribution compared to 450NGBD. We also limited the time points, following the 3R rules. The distribution of radiolabeled nanoparticles was generally similar to that in healthy mice. Relatively high tracer concentrations were observed in the liver and the spleen. Full data (tissue distribution) are available in Additional File S1: Tables S4 and S5.

**Figure 10:**
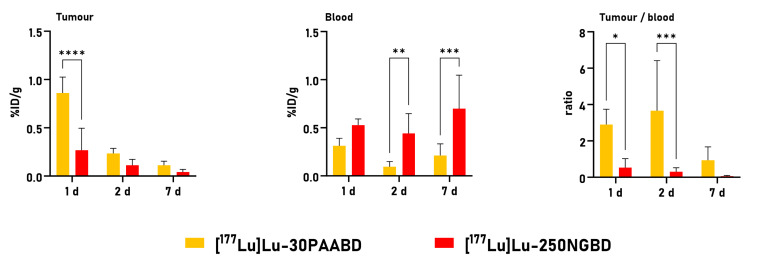
Tumor and blood uptake of [^177^Lu]Lu-250NGBD and [^177^Lu]Lu-30PAABD at 1, 2, and 7days post-injection (p.i.) in BALB/c NUDE mice engrafted s.c. with PC-3 cells. Tumor-to-blood ratio (TBR) for PC-3 tumors, the mean ± SEM (*n* = 3–5). Results are expressed as mean ± SEM. *p* < 0.05 is considered statistically significant, * *p* < 0.05, ** *p* < 0.005, *** *p* < 0,0005, **** *p* < 0.0001. %ID/g—percentage of injected dose per gram of selected organ or tissue.

We can see from Figure 10 that the tumor uptake was very low and did not exceed 1%ID/g for both radionanoparticles.

In the mice engraftment experiment, the tumor-to-blood ratio (TBR) was evaluated as a powerful tool for the semiquantitative assessment of [^177^Lu]Lu-PAABD/[^177^Lu]Lu-NGBD tumor uptake. The statistical analysis revealed that the more favorable TBR was for 30PAA at time points of 1 and 2 days. However, the obtained value was not satisfactory. As a final result, the high total binding of PAABD/NGBD to GRPR receptors in the PC-3 cells was not confirmed by this in vivo study using tumor-bearing mice.

Our study’s results align with the summary of the literature data presented by Cheng et al. [79]. In the review article, based on information derived from 232 data sets, they showed that a median of 0.7% of the injected dose (ID) of the nanoparticles reached the tumor. The median delivery efficiency has not improved in the past ten years. In other words, only 7 out of 1000 tested nanoparticles entered a solid tumor in a murine model [80].

One of the reasons that nanoparticles have very high retention in the liver is, according to Li et al. [81], slow blood flow. They used their poly(acrylic acid) nanoparticles to deliver norepinephrine and thus increase the blood flow in the liver. They found a significant increase in nanoparticle delivery to the solid tumor. This could be one of the directions for further improvement in our nanoplatform. They used chemical synthesis to obtain nanoparticles, and, with our radiation-derived nanoparticles, we could improve their concept and provide a higher-quality nanocarrier. 

## 4. Conclusions

This article presents a complete study concerning the development of a unique platform of nanocarriers based on poly(acrylic acid) for the targeted delivery of radioactive isotopes. We employed radiation crosslinking for the synthesis of polymer nanogels of various molecular weights corresponding to the diameter. Then, the obtained nanogels were functionalized with the targeting peptide coupled with a chelating moiety (DOTA). In the next step, we optimized the radiolabeling process and performed a basic systematic study of the biological effects, including cellular uptake, tissue distribution, and excretion. As a result of the in vitro studies, the developed nanoparticles showed strong potential for theranostic purposes. However, the distribution evaluated in vivo, crucial for future application, was unsatisfactory in both types of mice.

The results indicate that the nanoparticles developed in this form require further intensive investigation for improved biodistribution and clearance before potential clinical application in prostate theranostics. Studies in healthy mice have revealed that, consistently with many other studies on targeted nanosystems, a significant portion of the nanocarrier was retained in the liver and spleen; hence, it might be beneficial to repurpose the current system for liver-targeted delivery while continuing the optimization of the protocols for more promising results with respect to prostate cancer. Hence, the obtained results do not preclude research on the platform of nanoparticles obtained from radiation crosslinking and functionalized with targeting peptides and chelators for radioisotopes. Further studies are needed to identify the factors that may lead to more satisfactory results—for instance, using still smaller nanogels than studied here, or modifying the carrier’s chemical structure, charge density, or physical structure. 

## Figures and Tables

**Table 1:** Characteristics of the nanogel production protocols, specifying the concentration of poly(acrylic acid) and absorbed radiation dose for nanogels based on polymers with different nominal molecular weights (250 kDa and 450 kDa). The dose unit—1 kGy—corresponds to 1 kJ/kg.

	250NG	450NG
Concentration [mmol of monomer units/L]	10.0	17.5
Total absorbed dose [kGy]	6.18	7.97

**Table 2:** Conditions for the multistep EDC/NHS reactions, including the pH, buffers, and temperature during the conjugation of nanogels with targeting ligand.

Reaction Scheme	pH at Activation	Buffer Used	pH at Conjugation	Buffer Used	Temperature
M1	not controlled	-(pure water)	7.4	PBS	4 °C
M2			8.2	0.1 M TES buffer	
M3	pH 5.5	0.1 M MES buffer	7.4	PBS	
M4			8.2	0.1 M TES buffer	
M5	pH 6.3	0.1 M MES buffer	7.4	PBS	
M6			8.2	0.1 M TES buffer	

**Table 3:** Conditions for the one-pot EDC/NHS reactions, including the pH, buffers, and temperature during the conjugation of nanogels with targeting ligand.

Reaction Scheme	pH at Activation	Buffer Used	Temperature
EN1	pH 5.5	0.1 M MES buffer	4 °C
EN2	pH 6.3		
EN3	pH 7.4	PBS	

**Table 4:** Conditions for the one-pot DMTMM reactions, including the pH, buffers, and temperature during the conjugation of nanogels with targeting ligand.

Reaction Scheme	pH at Activation	Buffer Used	Temperature
D1	pH 5.5	0.1 M MES buffer	4 °C
D2	pH 6.3		
D3	pH 7.4	PBS	

**Table 5:** Primers used for real-time quantitative polymerase chain reaction. *GRPR*—gastrin-releasing peptide receptor, *RPLP0*—ribosomal protein P0, *RPS17*—ribosomal protein S17, *H3F3A*—histone H3.3A.

Gene	Sequence (5′-3′)	Product Size (bp)
*GRPR*	For TGATCCAGAGTGCTTACAATC Rev CGAACAGGCCCACAAACAC	111
*RPLP0*	For ACGGATTACACCTTCCCACTTGCTAAAAGGTC Rev AGCCACAAAGGCAGATGGATCAGCCAAG	69
*RPS17*	For AAGCGCGTGTGCGAGGAGATCG Rev TCGCTTCATCAGAT GCGTGACATAACCTG	87
*H3F3A*	For AGGACTTTAAAAGATCTGCGCTTCCAGAG Rev ACCAGATAGGCCTCACTTGCCTCCTGC	74

**Table 6:** Antibodies used for Western blotting.

Protein	Primary Antibody	Secondary Antibody
GAPDH	Mouse monoclonal anti-GAPDH antibody (overnight at 4 °C, 1:10,000 dilution; sc-59540, Santa Cruz Biotechnology Inc., Dallas, TX, USA)	Alkaline phosphatase-conjugated goat anti-mouse polyclonal antibody (4 h at 4 °C, 1:15,000 dilution; A3688, Sigma Aldrich, St. Louis, MO, USA)
GRPR	Rabbit polyclonal anti-GRPR antibody (overnight at 4 °C, 1:1000 dilution; PA5-33833, Thermo Fisher Scientific Inc., Waltham, MA, USA)	Alkaline phosphatase-conjugated goat anti-rabbit polyclonal antibody (4 h at 4 °C, 1:15,000 dilution; A3687, Sigma Aldrich, St. Louis, MO, USA)

**Table 7:** Characteristics of the nanogels used for the synthesis of targeted nanocarriers.

	250NG	450NG
Weight-average molecular weight [kDa]	239 ± 3	1449 ± 21
Hydrodynamic diameter [nm]	67 ± 5	124 ± 16

**Table 8:** The stability study of [^177^Lu]Lu-NGBD/[^177^Lu]Lu-PAABD in AAB and HS expressed as radiochemical purity (%). The value in brackets represents the samples before purifying.

RCP [%] Labeling with ^177^Lu						
		1 h	1 Day	4 Days	7 Days	14 Days
30PAABD	AAB	100.0 ± 0.00 (96.1 ± 1.72)	99.1 ± 0.39	98.9 ± 1.27	97.3 ± 1.55	96.0 ± 0.70
	HS	99.0 ± 1.87	95.4 ± 1.47	87.1 ± 4.66	86.9 ± 6.45	n.d.
250NGBD	AAB	99.5 ± 0.10	98.8 ± 1.00	97.4 ± 0.36	96.0 ± 2.93	96.0 ± 0.06
	HS	98.9 ± 0.43	98.6 ± 0.00	n.d.	96.9 ± 0.20	87.8 ± 1.81
450NGBD	AAB	100.0 ± 0.00	100.0 ± 0.00	98.5 ± 1.12	97.7 ± 0.15	99.3 ± 0.15
	HS	n.d.	98.9 ± 0.78	n.d.	94.7 ± 1.22	93.0 ± 2.03

n.d.—not detected.

**Table 9:** The stability study of [^90^Y]Y-PAA in AAB and HS expressed as radiochemical purity (%). The value in brackets represents the samples before purifying.

RCP [%] Labeling with ^90^Y						
		1 h	1 Day	4 Days	7 Days	14 Days
30PAABD	AAB	99.8 ± 0.24 (94.5 ± 1.36)	97.6 ± 2.37	98.4 ± 0.88	95.4 ± 0.63	94.6 ± 0.20
	HS	95.9 ± 1.17	94.0 ± 0.25	91.3 ± 0.68	93.1 ± 1.21	n.d.
250NGBD	AAB	98.2 ± 1.32	98.8 ± 0.60	98.4 ± 0.36	98.2 ± 1.71	97.9 ± 0.96
	HS	93.2 ± 0.14	97.4 ± 0.00	n.d.	95.8 ± 2.67	96.0 ± 0.78
450NGBD	AAB	100 ± 0.00	99.4 ± 0.00	99.2 ± 0.06	98.3 ± 0.17	99.0 ± 0.26
	HS	99.8 ± 0.00	99.7 ± 0.38	97.8 ± 1.20	92.9 ± 0.25	96.3 ± 1.37

n.d.—not detected.

**Table 10:** Value of the Spearman’s rank correlation coefficient (r_S_) considering in vivo.

	450NGBD	250NGBD	30PAABD
*p*-value	0.8028	0.0222	0.0167
r_s_	−0.1429	−0.9276	−1.000

## Data Availability

Data are contained within the article.

## Supplementary Materials

The following supporting information can be downloaded at: https://www.mdpi.com/article/10.3390/cancers15235646/s1, Additional File S1: Optimization of PAA nanogel synthesis; Figure S1: Weight-average molecular weight (M_w_) of PAA structures as a function of absorbed dose for electron-beam-irradiated samples in Ar-saturated solutions: (a) 250PAA; (b) 450PAA. Irradiation parameters: dose per pulse 0.90 kGy, pulse duration 4 µs, pulse frequency 0.5 Hz. Solution properties during irradiation: pH 2.0, maintained with HClO_4_, saturated with Ar, PAA concentrations (as mM of monomer units per L) given in the graphs; Figure S2: Radius of gyration (R_g_) of PAA structures as a function of absorbed dose for electron-beam-irradiated samples in Ar-saturated solutions: (a) 250PAA; (b) 450PAA. Irradiation parameters: dose per pulse 0.90 kGy, pulse duration 4 µs, pulse frequency 0.5 Hz. Solution properties during irradiation: pH 2.0, maintained with HClO_4_, saturated with Ar. PAA concentrations (as mM of monomer units per L) given in the graphs; Figure S3: Density of PAA coils, defined by the ratio of M_w_ to the volume of a sphere of the radius R_g_, as a function of absorbed dose for electron beam irradiated samples in Ar-saturated solutions: (a) 250PAA; (b) 450PAA. Irradiation parameters: dose per pulse 0.90 kGy, pulse duration 4 µs, pulse frequency 0.5 Hz. Solution properties during irradiation: pH 2.0, maintained with HClO_4_, saturated with Ar. PAA concentrations (as mM of monomer units per L) given in the graphs; Additional File S2: Physiological distribution data; Table S1: Results of biodistribution of the [^177^Lu]Lu-30PAA (molecular weight 30 kDa) in BALB/c mice (%ID/g, mean ± SD, *n* = 5); Table S2: Results of biodistribution of the [^177^Lu]Lu-PAA (molecular weight 250 kDa) in BALB/c mice (%ID/g, mean ± SD, *n* = 5); Table S3: Results of biodistribution of the [^177^Lu]Lu-PAA (molecular weight 450 kDa) in BALB/c mice (%ID/g, mean ± SD, *n* = 5); Table S4: Results of biodistribution of the [^177^Lu]Lu-PAA (molecular weight 30 kDa) in BALB/c NUDE mice (%ID/g, mean ± SD, *n* = 5); Table S5: Results of biodistribution of the [^177^Lu]Lu-PAA (molecular weight 250 kDa) in BALB/c NUDE mice (%ID/g, mean ± SD, *n* = 5); Additional File S3: Western blot raw data used for analysis. References [37,82] are cited in the supplementary materials.
