# Supplementary material for: Towards Cancer Nanoradiopharmaceuticals—Radioisotope Nanocarrier System for Prostate Cancer Theranostics Based on Radiation-Synthesized Polymer Nanogels
Source: Cancers (Basel). 2023 Nov 29;15(23):5646. doi: 10.3390/cancers15235646 (PMC10705749; doi:10.3390/cancers15235646)
Supplement: Supplementary file 1 [file cancers-15-05646-s001.zip › cancers-2666868-supplementary.pdf]

Additional File S1: Optimization of PAA nanogels synthesis

Formation of nanogels requires the internal (intramolecular) crosslinking of the polymer coils with the number of crosslinks sufficient to stabilize the structure and therefore increase its internal density. Conditions necessary for the formation of nanogels include sufficiently high dose of pulse-delivered ionizing radiation and low concentration of the compact polymer coils [1]. However, extremely low concentration of the polymer and high irradiation doses in pulsed radiolysis regime are impractical, therefore there was a need for optimized conditions. In order to choose the conditions that would yield a viable process and product with most beneficial properties, various concentrations of polymer and multiple doses of radiation were screened in preparative pulsed radiolysis of poly(acrylic acid) (PAA). Polymer samples with two distinct nominal molecular weights: 250 kDa (250PAA) (Sigma Aldrich, Cat. No. 416002, Lot: DTBG9347 Steinheim, Germany) and 450 kDa (450PAA) (Polysciences Inc., Cat. No. 03312-100, Lot: 697844; Hirschberg an der Bergstrasse, Germany) were examined.

Upon systematic analysis, influence of the polymer concentration and the total absorbed dose of radiation on the molecular weight of polymer structures, radius of gyration and coil density was determined (Figures S1–S3). Based on the collected results, it was concluded that in case of 450PAA, the most preferable conditions for internal crosslinking were achieved at a concentration of 17.5 mM and upon irradiation with a dose of ca. 8.5 kGy; it was possible to obtain the most compact nanostructures with highest coil density. In case of 250PAA, the most favourable conditions for internal crosslinking were found for the 10 mM solution, where molecular weights are almost unchanged, and around 6.3 kGy, where the most compact structures were obtained.

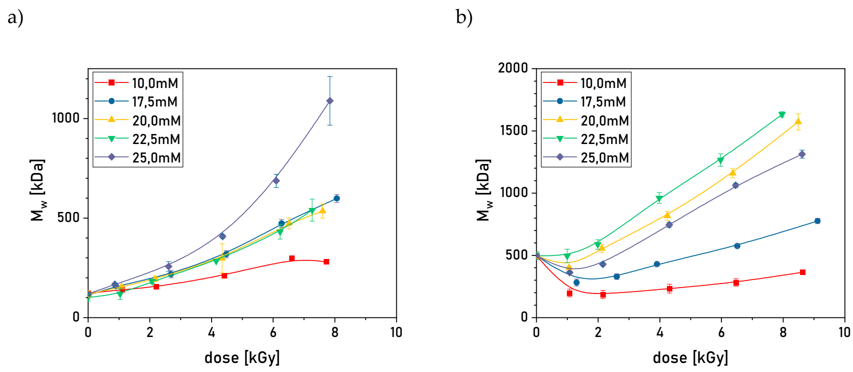

Figure S1. Weight-average molecular weight (M_w) of PAA structures as a function of absorbed dose for electron-beam-irradiated samples in Ar-saturated solutions: a) 250PAA; b) 450PAA. Irradiation parameters: dose per pulse 0.90 kGy, pulse duration 4 µs, pulse frequency 0.5 Hz. Solution properties during irradiation: pH 2.0, maintained with $HClO_4$, saturated with Ar, PAA concentrations (as mM of monomer units per L) given in the graphs.

a) b)

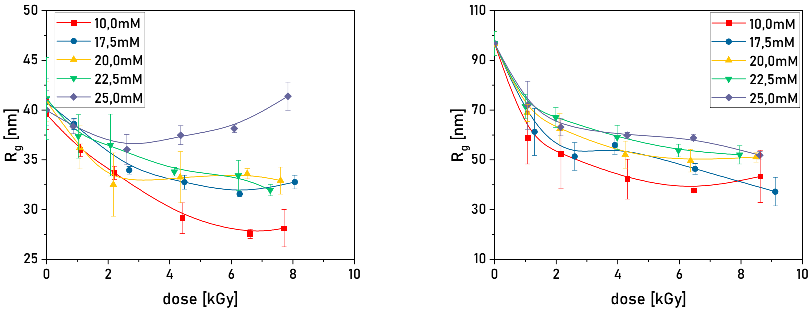

Figure S2. Radius of gyration (R_g) of PAA structures as a function of absorbed dose for electron-beam-irradiated samples in Ar- saturated solutions: a) 250PAA; b) 450PAA. Irradiation parameters: dose per pulse 0.90 kGy, pulse duration 4 μs, pulse frequency 0.5 Hz. Solution properties during irradiation: pH 2.0, maintained with $HClO_4$, saturated with Ar. PAA concentrations (as mM of monomer units per L) given in the graphs.

a) b)

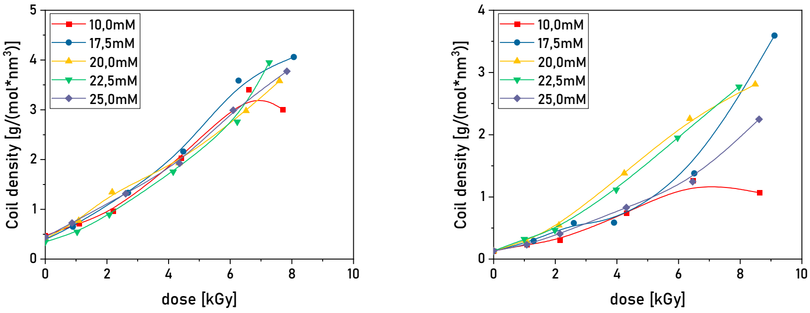

Figure S3. Density of PAA coils, defined by the ratio of M_w to the volume of a sphere of the radius R_g, as a function of absorbed dose for electron beam irradiated samples in Ar-saturated solutions: a) 250PAA; b) 450PAA. Irradiation parameters: dose per pulse 0.90 kGy, pulse duration 4 μs, pulse frequency 0.5 Hz. Solution properties during irradiation: pH 2.0, maintained with $HClO_4$, saturated with Ar. PAA concentrations (as mM of monomer units per L) given in the graphs.

Additional File S2: Physiological Distribution data.

Table S1. Results of biodistribution of the [177Lu]Lu-30PAA (molecular weight 30 kDa) in BALB/c mice (%ID/g, mean ± SD, n = 5).

organ	4h	1 day	2 days	4 days	7 days
blood	1.05 ± 0.24	1.35 ± 1.13	0.63 ± 0.36	0.44 ± 0.18	0.37 ± 0.25
thyroid	0.84 ± 0.07	1.08 ± 0.50	0.70 ± 0.22	0.56 ± 0.31	0.97 ± 0.40
heart	1.15 ± 0.23	1.30 ± 0.48	0.67 ± 0.11	0.44 ± 0.23	0.28 ± 0.25
lung	2.86 ± 0.43	2.37 ± 0.29	1.38 ± 0.34	0.92 ± 0.27	0.57 ± 0.18
liver	57.71 ± 5.41	45.75 ± 3.60	36.87 ± 4.97	27.00 ± 2.40	18.85 ± 2.50
spleen	16.08 ± 2.63	21.68 ± 2.81	18.35 ± 2.33	11.35 ± 2.92	7.64 ± 4.67
kidneys	22.43 ± 0.80	18.03 ± 3.44	10.82 ± 0.38	6.56 ± 1.21	2.47 ± 1.32
small intestine	1.06 ± 0.12	0.97 ± 0.07	0.67 ± 0.11	0.41 ± 0.08	0.31 ± 0.07
large intestine	0.82 ± 0.11	0.89 ± 0.20	0.66 ± 0.17	0.31 ± 0.05	0.20 ± 0.06
stomach	1.69 ± 0.11	1.61 ± 0.25	0.93 ± 0.11	0.55 ± 0.09	0.34 ± 0.17
femur	3.73 ± 0.69	4.90 ± 1.25	2.80 ± 0.35	2.31 ± 0.42	2.32 ± 1.11
muscle	0.34 ± 0.05	0.36 ± 0.13	0.30 ± 0.07	0.16 ± 0.1	0.36 ± 0.14
urine [%ID]	11.37 ± 0.88	20.47 ± 6.19	34.95 ± 3.05	59.87 ± 1.95	72.57 ± 2.16

Table S2. Results of biodistribution of the [177Lu]Lu-PAA (molecular weight 250 kDa) in BALB/c mice (%ID/g, mean ± SD, n = 5).

organ	2h	4h	1 day	2 days	4 days	7 days
blood	1.11 ± 0.28	1.11 ± 0.44	1.00 ± 0.31	0.75 ± 0.13	0.84 ± 0.42	0.67 ± 0.22
thyroid	1.10 ± 0.43	0.81 ± 0.39	0.52 ± 0.36	0.56± 0.52	0.25 ± 0.11	1.15 ± 0.10
heart	2.36 ± 0.78	2.31 ± 0.77	1.47 ± 0.57	1.12 ± 0.35	0.86 ± 0.08	0.62 ± 0.23
lung	2.54± 0.78	2.26 ± 0.44	0.25 ± 0.67	1.18 ± 0.28	1.18 ± 0.16	0.81 ± 0.27
liver	63.99 ± 5.62	55.83 ± 5.22	55.51 ± 5.75	54.34 ± 1.99	34.04 ± 3.49	21.17 ± 1.24
spleen	15.63 ± 5.30	13.56 ± 4.25	20.57 ± 4.02	18.16 ± 6.06	10.81 ± 1.66	8.49 ± 1.50
kidneys	3.69 ± 0.47	3.06 ± 0.57	2.72 ± 0.48	2.34 ± 0.60	1.45 ± 0.21	0.94 ± 0.25
small intestine	0.26 ± 0.07	0.37 ± 0.19	0.25 ± 0.06	0.22 ± 0.08	0.06 ± 0.02	0.06 ± 0.03
large intestine	0.31 ± 0.09	0.30 ± 0.11	0.45 ± 0.16	0.62 ± 0.16	0.13 ± 0.03	0.12 ± 0.04
stomach	0.46 ± 0.10	0.33 ± 0.09	0.38 ± 0.13	0.37 ± 0.23	0.32 ± 0.27	0.42 ± 0,38
femur	3.16 ± 0.85	2.68 ± 0.56	2.89 ± 0.35	3.14 ± 0.72	2.62 ± 0.40	2.21 ± 1.09
muscle	0.59 ± 0.27	0.43 ± 0.11	0.44 ± 0.14	0.30 ± 0.29	0.34 ± 0.07	0.29 ± 0.18
urine [%ID]	3.75 ± 0.36	5.20 ± 0.82	13.69 ± 1.72	26.78 ± 1.33	44.68 ± 2.52	61.46 ± 2.28

Table S3. Results of biodistribution of the [177Lu]Lu-PAA (molecular weight 450 kDa) in BALB/c mice (%ID/g, mean ± SD, n = 5).

organ	2h	4h	1 day	2 days	4 days	7 days
blood	1.72±1.36	0.67±0.12	0.88±0.41	0.40±0.14	0.55±0.26	0.44±0.20
thyroid	0.75±0.43	0.77±0.52	0.64±0.20	0.79±0.61	0.34±0.18	0.25±0.15
heart	3.15±2.87	1.81±1.23	1.95±1.24	1.09±0.58	0.44±0.10	0.19±0.08
lung	4.32±3.04	3.71±1.48	2.63±0.57	1.83±0.63	1.12±0.27	0.61±0.10
liver	90.24±12.40	73.65±5.85	82.35±6.88	54.48±6.98	36.25±2.15	26.93±4.82

spleen	26.52±6.11	24.96±2.66	34.68±16.94	21.72±5.30	22.73±4.92	18.18±5.73
kidneys	1.68±0.56	1.66±0.23	1.57±0.24	1.41±0.31	0.79±0.12	0.59±0.12
small intestine	0.30±0.20	0.34±0.14	0.52±0.12	0.50±0.28	0.16±0.04	0.13±0.07
large intestine	0.36±0.26	0.46±0.21	1.65±0.33	1.36±0.35	0.16±0.02	0.05±0.02
stomach	0.32±0.15	0.36±0.06	0.45±0.07	0.22±0.03	0.15±0.03	0.13±0.03
femur	2.05±0.56	2.10±0.24	2.50±0.57	1.40±0.74	1.60±0.39	2.01±0.66
muscle	0.56±0.41	0.46±0.15	0.58±0.25	0.37±0.20	0.20±0.10	0.08±0.06
urine [%ID]	2.37±0.41	4.50±0.64	11.40±2.38	24.60±4.28	b.d.	

Table S4. Results of biodistribution of the [177Lu]Lu-PAA (molecular weight 30 kDa) in BALB/c NUDE mice (%ID/g, mean ± SD, n = 5).

organ	1 day	4 days	7 days
blood	0.31±0.08	0.09 ± 0.06	0.18 ± 0.08
thyroid	1.13 ± 0.33	0.59 ± 0.30	0.42 ± 0.25
heart	0.91 ± 0.10	0.49 ± 0.10	0.35 ± 0.13
lung	1.45 ± 0.29	0.43 ± 0.08	0.37 ± 0.12
liver	31.52 ± 3.03	16.90 ± 2.63	18.97 ± 2.12
spleen	15.24 ± 1.98	6.75 ± 1.20	7.40 ± 1.90
kidneys	14.47 ± 1.93	4.12 ± 0.25	3.10 ± 0.36
small intestine	1.66 ± 0.24	1.13 ± 0.12	0.99 ± 0.18
large intestine	1.34 ± 0.33	0.47 ± 0.06	0.47 ± 0.04
stomach	1.43 ± 0.27	0.59 ± 0.10	0.35 ± 0.19
femur	4.23 ± 0.59	2.21 ± 0.60	2.44 ± 0.47
muscle	0.48 ± 0.04	0.31 ± 0.11	0.14 ± 0.09
tumour	0.86 ± 0.16	0.23 ± 0.05	0.11 ± 0.04
urine [%ID]	29.96 ± 0.82	59.79 ± 1.39	61.24 ± 1.15

Table S5. Results of biodistribution of the [177Lu]Lu-PAA (molecular weight 250 kDa) in BALB/c NUDE mice (%ID/g, mean ± SD, n = 5).

organ	4 h	1 day	4 days	7 days
blood	0.60 ± 0.22	0.53 ± 0.06	0.61 ± 0.57	0.70 ± 0.35
thyroid	0.29 ± 0.19	0.49 ± 0.13	0.47 ± 0.28	0.43 ± 0.19
heart	0.70 ± 0.32	1.18 ± 0.24	0.58 ± 0.13	0.43 ± 0.13
lung	1.87 ± 0.26	1.61 ± 0.44	1.26 ± 0.67	0.83 ± 0.27
liver	57.07 ± 8.92	45.33 ± 5.13	54.15 ± 6.84	35.81 ± 3.10
spleen	15.59 ± 6.37	25.44 ± 3.24	31.74 ± 9.63	15.12 ± 4.93
kidneys	0.86 ± 0.34	2.25 ± 0.29	1.43 ± 0.69	1.17 ± 0.07
small intestine	0.28 ± 0.02	0.26 ± 0.10	0.18 ± 0.04	0.16 ± 0.08
large intestine	0.41 ± 0.17	0.21 ± 0.08	0.27 ± 0.09	0.12 ± 0.03
stomach	0.39 ± 0.21	0.23 ± 0.04	0.20 ± 0.06	0.11 ± 0.05
femur	2.94 ± 0.90	3.36 ± 0.33	3.06 ± 0.82	2.21 ± 0.46
muscle	0.26 ± 0.12	0.36 ± 0.07	0.04 ± 0.03	0.21 ± 0.13
tumour	0.25 ± 0.16	0.27 ± 0.23	0.11 ± 0.06	0.04 ± 0.03
urine [%ID]	4.36 ± 0.47	11.30 ± 1.59	28.43 ± 4.94	47.79 ± 1.14

Additional File S3: Western blot raw data used for analysis.

The figures present the original results from Western blot analysis. Each band represents the biological replicate of the probe. The technical replicates were made on separates gels. MCF-7 cell line was used as a positive control for the analysis, based on the literature [2] and availability.

Commented [KK1]: Beata: prośba o wrzucenie tu referencji: https://www.spandidos-publications.com/10.3892/mco.2012.7

Blot 1

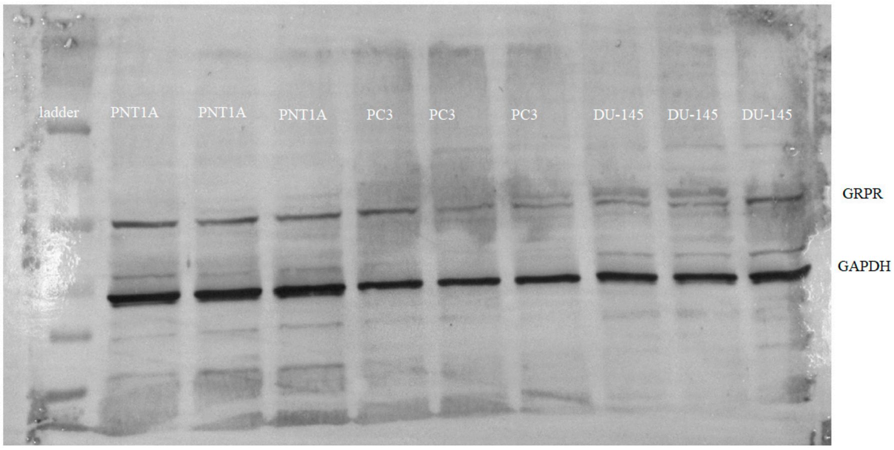

Blot 2

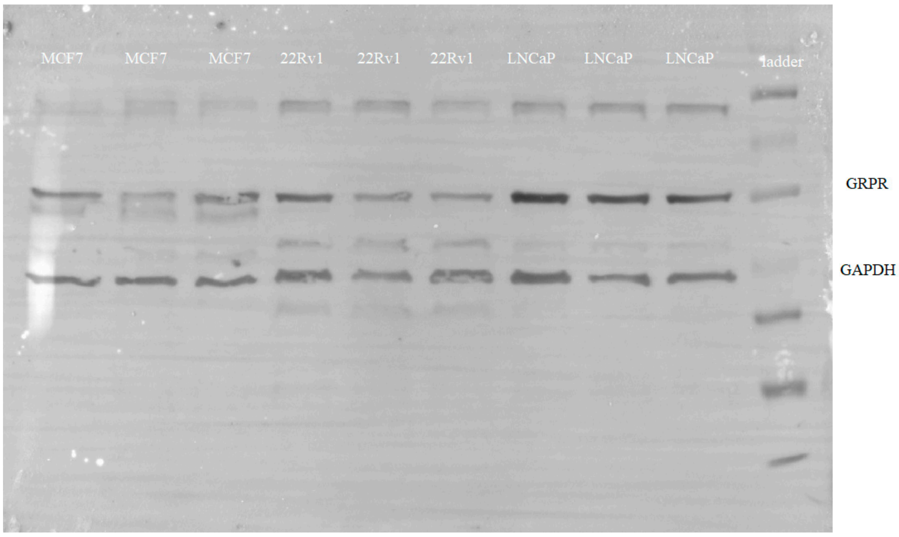

Blot 3

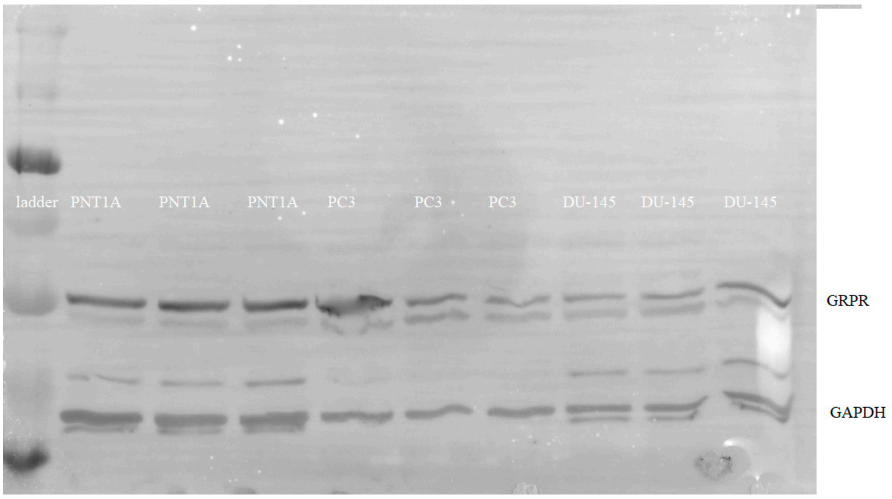

ladder
PNT1A
PNT1A
PNT1A
PC3
PC3
PC3
DU-145
DU-145
DU-145
GRPR
GAPDH

Blot 4

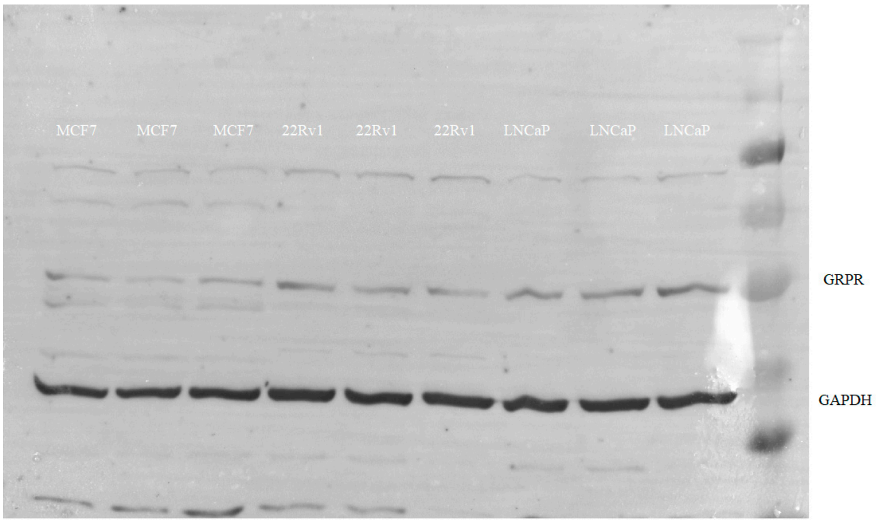

MCF7
MCF7
MCF7
22Rv1
22Rv1
22Rv1
LNCaP
LNCaP
LNCaP
GRPR
GAPDH

Blot 5

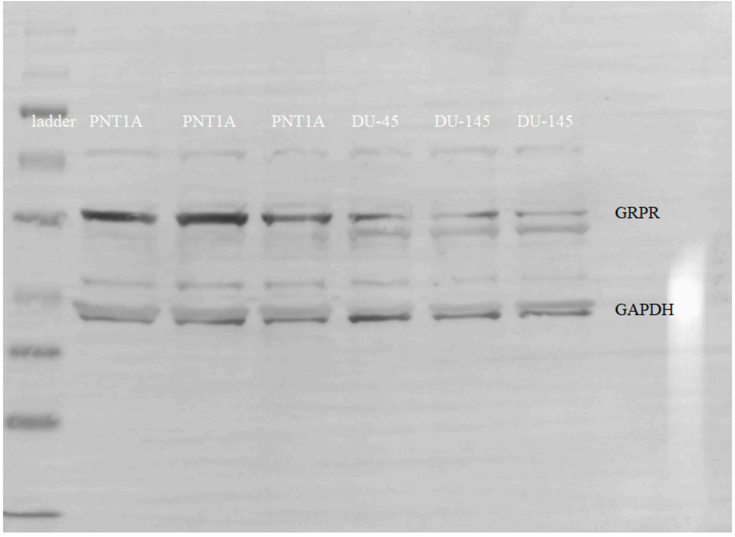
ladder
PNT1A
PNT1A
PNT1A
DU-45
DU-145
DU-145
GRPR
GAPDH

Blot 6

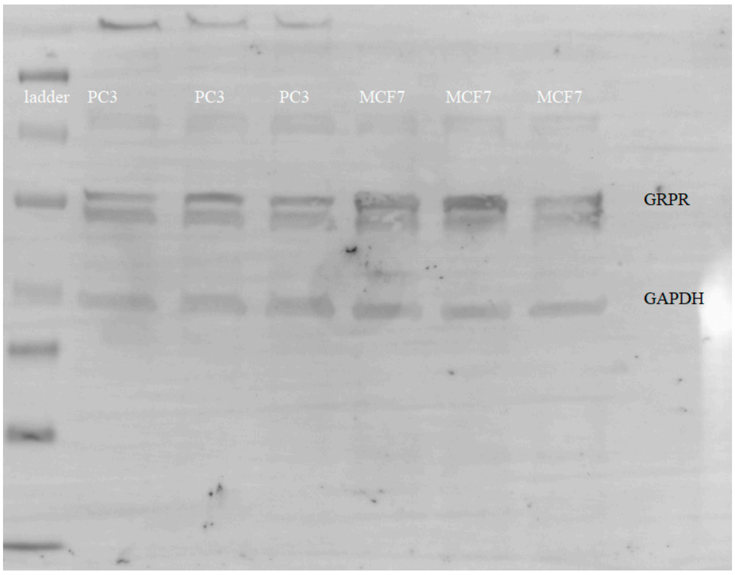
ladder
PC3
PC3
PC3
MCF7
MCF7
MCF7
GRPR
GAPDH

Blot 7

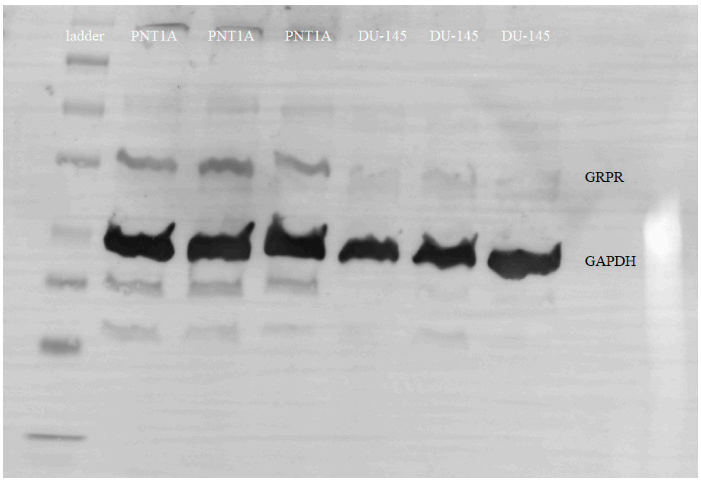

Blot 8

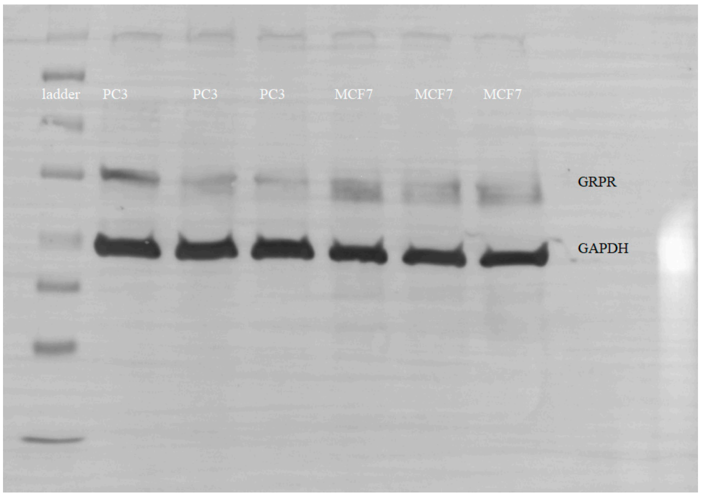

References

1. Matusiak, M.; Kadlubowski, S.; Ulanski, P. Radiation-induced synthesis of poly(acrylic acid) nanogels. *Radiat. Phys. Chem.* **2018**, *142*, 125–129.
2. Cornelio, D.B.; De Farias, C.B.; Prusch, D.S.; Heinen, T.E.; Dos Santos, R.P.; Abujamra, A.L.; Schwartsmann, G.; Roesler, R. Influence of GRPR and BDNF/TrkB signaling on the viability of

breast and gynecologic cancer cells. *Mol. Clin. Oncol.* **2013**, *1*, 148–152.
